# Repeated emergence and fitness heterogeneity of KPC-33 in ST11 *Klebsiella pneumoniae* under ceftazidime–avibactam pressure

**DOI:** 10.1128/aac.00767-26

**Published:** 2026-09-10

**Authors:** Fangling Du, Xinyue Dai, Yuxuan Liu, Chuwen Zhao, Hanxu Hong, Yun Cao, Dandan Wei, Yang Liu

**Affiliations:** 1 Department of Clinical Laboratory, The First Affiliated Hospital, Jiangxi Medical College, Nanchang University 47861, Nanchang, People's Republic of China; 2 School of Public Health, Jiangxi Medical College, Nanchang University 47861, Nanchang, People's Republic of China; 3 Jiangxi Medical Center for Critical Public Health Events, The First Affiliated Hospital, Jiangxi Medical College, Nanchang University 47861, Nanchang, People's Republic of China; Columbia University Irving Medical Center, New York, New York, USA

**Keywords:** *Klebsiella pneumoniae*, KPC-33, ceftazidime–avibactam, resistance evolution, fitness cost

## Abstract

Ceftazidime–avibactam (CZA) is an important therapeutic option for infections caused by *Klebsiella pneumoniae* carbapenemase (KPC)-producing *Klebsiella pneumoniae*. However, CZA exposure also selects for emergent KPC variants. Their *in vivo* evolutionary patterns, fitness consequences, and underlying molecular mechanisms remain unclear. We performed a longitudinal multiomics analysis of 35 clonally related ST11 KPC-producing *K. pneumoniae* isolates collected from eight hospitalized patients during clinical follow-up, most of whom had received CZA therapy. Whole-genome sequencing, antimicrobial susceptibility testing, *in vitro* competition assays, enzyme kinetic analysis, and transcriptomic sequencing were used to systematically characterize the within-host evolutionary dynamics of KPC variants and the fitness heterogeneity of KPC-33. Multiple KPC variants were identified during longitudinal follow-up, among which KPC-33 was the most frequently detected. Among the seven patients who received CZA treatment, KPC-33 was detected in longitudinal isolates from four patients. It was also identified in patient P3, who had not received CZA, whereas other variants were only sporadically identified. Biochemical analysis showed that KPC-33 exhibited an altered kinetic profile relative to KPC-2, characterized by reduced catalytic turnover and altered substrate affinity. KPC-33 did not exhibit a uniform and pronounced fitness defect but instead showed marked strain-dependent heterogeneity. Strains with higher competitive fitness generally showed only limited transcriptional changes, whereas those with lower fitness were accompanied by broader transcriptional remodeling. In this longitudinal cohort, KPC-33 was repeatedly detected, predominantly under CZA-associated selective conditions. Its fitness consequences were clearly strain background dependent and may be associated with the extent of transcriptional remodeling. These findings provide new evidence for understanding the *in vivo* evolution of CZA resistance.

## INTRODUCTION

Carbapenem-resistant *Klebsiella pneumoniae* (CRKP) is one of the most threatening multidrug-resistant pathogens to global public health and is closely associated with severe hospital-acquired infections and high mortality (1). Epidemiological studies have shown that, among the various carbapenemases, *Klebsiella pneumoniae* carbapenemase (KPC) is one of the predominant resistance mechanisms. In China in particular, ST11 *K. pneumoniae*-producing KPC-2 has become the major epidemic clinical clone (2, 3). Ceftazidime–avibactam (CZA), a novel β-lactam/β-lactamase inhibitor combination, has potent antimicrobial activity against KPC-producing Enterobacterales and has become a first-line treatment for infections caused by KPC-2-producing CRKP (4).

With the widespread clinical use of CZA, resistance during therapy has become increasingly evident. Under antibiotic selective pressure, the *bla*_KPC_ gene can undergo point mutations, deletions, or insertions, leading to the emergence of new KPC variants and consequent CZA resistance (5, 6). Multiple KPC variants associated with CZA resistance have been identified to date, including KPC-33, KPC-44, KPC-112, and KPC-204 (7–10)

However, existing studies have mainly focused on case reports or molecular mechanisms. Although some studies have preliminarily described the distribution of KPC variants in clinical settings, their dynamic within-host evolutionary processes and fitness characteristics still lack systematic longitudinal investigation *in vivo* (11, 12).

Based on this, we consecutively enrolled eight patients infected with clonally related ST11 KPC-2-producing *K. pneumoniae* and longitudinally collected serial isolates throughout the entire treatment course. By integrating whole-genome sequencing, *in vitro* fitness assays, enzyme kinetic analysis, and transcriptomic sequencing, we systematically characterized the dynamic evolution of KPC-2 into different KPC variants under CZA treatment pressure, with a particular focus on the recurrent emergence of KPC-33 and its fitness heterogeneity. This study aimed to provide a new perspective for understanding the evolution of CZA resistance and to offer a scientific basis for optimizing antimicrobial treatment strategies in clinical practice.

## RESULTS

### Repeated emergence of KPC-33 during *in vivo* evolution

A total of eight hospitalized patients infected with clonally related ST11 *K. pneumoniae* carrying *bla*_KPC-2_ were included in this study, and 35 clinical isolates recovered at different time points during hospitalization were collected longitudinally. All patients were male, with a median age of 54 years (range, 27–69 years). The initial isolate from each patient harbored *bla*_KPC-2_ and was susceptible to CZA (minimum inhibitory concentration [MIC] = 4 mg/L).

**Fig 1:**
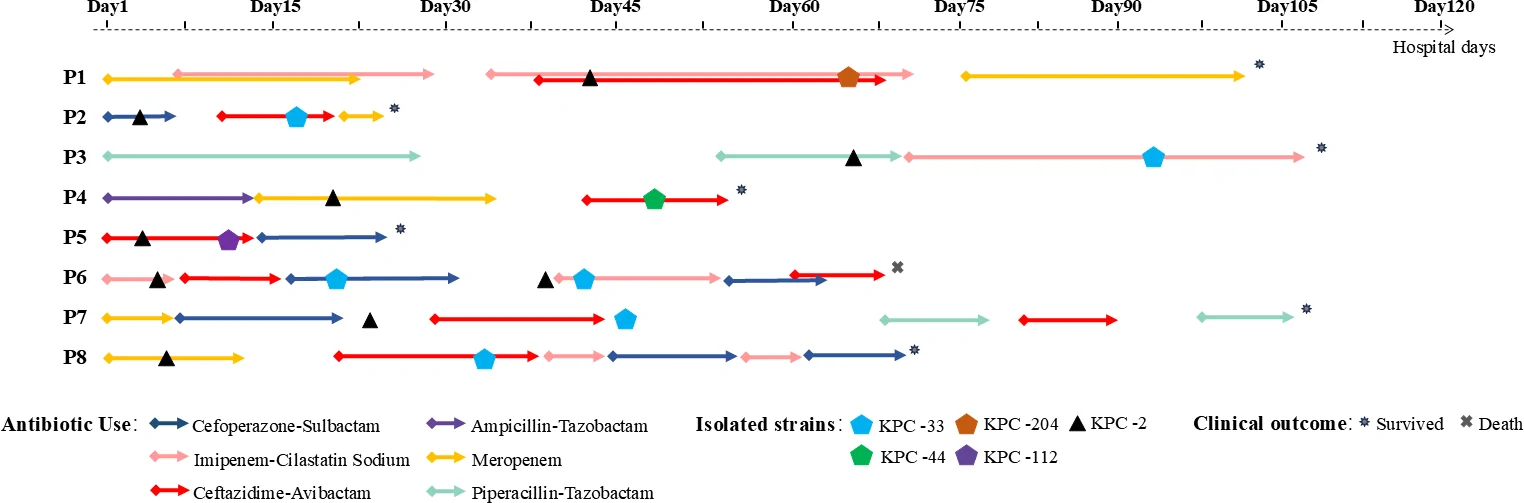
Longitudinal evolution of KPC genotypes during hospitalization. Colored lines denote antibiotic exposure; symbols indicate isolates with different KPC genotypes; and clinical outcomes are shown at the end of each timeline. Only the isolation time of the first KPC-2 isolate is shown for each patient.

Among the seven patients who underwent CZA therapy, KPC variants were first detected 5–17 days after treatment initiation. In contrast, patient P3 did not receive CZA but had been treated with piperacillin–tazobactam and aztreonam during hospitalization; one KPC-33 isolate was recovered from this patient on hospital day 91. (Fig. 1). Among the nine KPC variant isolates identified, six were KPC-33, accounting for 66.7% and representing the most frequently detected variant in this study. The remaining variants, including KPC-44, KPC-112, and KPC-204, were detected only sporadically. Notably, two KPC-33 variant isolates were recovered sequentially from patient P6.

Antimicrobial susceptibility testing showed that, compared with their respective parental KPC-2 isolates, all KPC variants exhibited increased MICs to CZA. The six KPC-33 isolates all had a CZA MIC of 16 mg/L, whereas the KPC-44, KPC-112, and KPC-204 isolates showed CZA MICs ranging from 64 to 256 mg/L (Table 1). Apart from the altered susceptibility to CZA, no consistent changes were observed in the susceptibility profiles of the variants to most of the other antimicrobial agents tested. Overall, KPC-33 was the most frequently detected KPC variant in this study and repeatedly emerged among longitudinal isolates from multiple patients, providing a clinical isolate basis for further analysis of its fitness effects.

**TABLE 1:** Characteristics and MICs of the 35 longitudinal CRKP isolates^*b*^*^,^*^*c*^

Patient	Sex	Strains	Collection data (month/day/year)	KPC variant	Amino and mutation^*a*^	IPM	MEM	CZA	TIM	TZP	CAZ	FEP	ATM	TGC
P1	M	P1-A1-W	10/14/2023	KPC-2	–	≥64	≥64	4/4	≥128	≥128	≥64	≥32	≥64	2
		P1-A2-W	10/13/2023	KPC-2	–	≥64	≥64	4/4	≥128	≥128	≥64	≥32	≥64	2
		P1-A3-W	10/14/2023	KPC-2	–	≥64	≥64	4/4	≥128	≥128	≥64	≥32	≥64	2
		P1-A4-W	10/17/2023	KPC-2	–	≥64	≥64	4/4	≥128	≥128	≥64	≥32	≥64	8
		P1-A5-W	10/20/2023	KPC-2	–	≥64	≥64	4/4	≥128	≥128	≥64	≥32	≥64	4
		P1-A6-W	10/23/2023	KPC-2	–	≥64	≥64	4/4	≥128	≥128	≥64	≥32	≥64	2
		P1-A7-V	10/27/2023	KPC-204	ins_269_KDD	8	32	>128/4	≥128	≥128	≥64	≥32	≥64	2
P2	M	P2-B1-W	6/23/2023	KPC-2	–	≥64	≥64	4/4	≥128	≥128	≥64	≥32	≥64	0.5
P2	M	P2-B2-V	7/4/2023	KPC-33	D179Y	1	4	16/4	≥128	≥128	≥64	≥32	≥64	0.5
		P2-B3-W	7/9/2023	KPC-2	–	≥64	≥64	4/4	≥128	≥128	≥64	≥32	≥64	0.5
P3	M	P3-C1-V	12/27/2023	KPC-33	D179Y	1	8	16/4	≥128	≥128	≥64	≥32	≥64	1
		P3-C2-W	10/25/2023	KPC-2	–	≥64	≥64	4/4	≥128	≥128	≥64	≥32	≥64	1
P4	M	P4-D1-W	8/2/2024	KPC-2	–	≥64	≥64	8/4	≥128	≥128	≥64	≥32	≥64	2
		P4-D2-W	8/22/2024	KPC-2	–	≥64	≥64	4/4	≥128	≥128	≥64	≥32	≥64	1
		P4-D3-V	8/29/2024	KPC-44	ins_270_KDDKH SEAVYTRAPN	32	32	64/4	≥128	≥128	≥64	≥32	≥64	1
P5	M	P5-E1-W	8/28/2024	KPC-2	–	≥64	≥64	4/4	≥128	≥128	≥64	≥32	≥64	1
		P5-E2-V	9/6/2024	KPC-112	del_166-167_LE, del_242-243_GT	0.25	2	>128/4	≥128	≥128	≥64	≥32	≥64	2
		P5-E3-W	9/13/2024	KPC-2	–	≥64	≥64	4/4	≥128	≥128	≥64	≥32	≥64	2
P6	M	P6-F1-W	9/27/2024	KPC-2	–	≥64	≥64	4/4	≥128	≥128	≥64	≥32	≥64	1
		P6-F2-V	10/18/2024	KPC-33	D179Y	0.25	16	16/4	≥128	≥128	≥64	≥32	≥64	1
		P6-F3-W	10/23/2024	KPC-2	–	≥64	≥64	4/4	≥128	≥128	≥64	≥32	≥64	1
		P6-F4-W	11/10/2024	KPC-2	–	≥64	≥64	4/4	≥128	≥128	≥64	≥32	≥64	1
		P6-F5-W	11/22/2024	KPC-2	–	≥64	≥64	4/4	≥128	≥128	≥64	≥32	≥64	1
		P6-F6-V	10/29/2024	KPC-33	D179Y	1	2	16/4	≥128	≥128	≥64	≥32	≥64	1
P7	M	P7-G1-W	11/3/2024	KPC-2	–	≥64	≥64	4/4	≥128	≥128	≥64	≥32	≥64	1
		P7-G2-W	11/7/2024	KPC-2	–	≥64	≥64	4/4	≥128	≥128	≥64	≥32	≥64	1
		P7-G3-V	11/21/2024	KPC-33	D179Y	0.5	2	16/4	≥128	≥128	≥64	≥32	≥64	2
		P7-G4-W	12/17/2024	KPC-2	–	≥64	≥64	4/4	≥128	≥128	≥64	≥32	≥64	1
		P7-G5-W	12/24/2024	KPC-2	–	≥64	≥64	4/4	≥128	≥128	≥64	≥32	≥64	1
		P7-G6-W	2/11/2024	KPC-2	–	≥64	≥64	4/4	≥128	≥128	≥64	≥32	≥64	1
P8	M	P8-H1-W	9/4/2024	KPC-2	–	≥64	≥64	4/4	≥128	≥128	≥64	≥32	≥64	1
		P8-H2-W	9/9/2024	KPC-2	–	≥64	≥64	4/4	≥128	≥128	≥64	≥32	≥64	2
		P8-H3-W	9/12/2024	KPC-2	–	≥64	≥64	4/4	≥128	≥128	≥64	≥32	≥64	2
		P8-H4-V	9/29/2024	KPC-33	D179Y	0.5	1	16/4	≥128	≥128	≥64	≥32	≥64	2
		P8-H5-W	10/12/2024	KPC-2	–	≥64	≥64	4/4	≥128	≥128	≥64	≥32	≥64	2

^*a*^ Amino acid alterations are described using the standard one-letter amino acid code; del, deletion; ins, insertion; –, separators.

^*b*^ ATM, aztreonam; CAZ, ceftazidime; CZA, ceftazidime–avibactam; FEP, cefepime; IPM, imipenem; MEM, meropenem; TIM, ticarcillin-clavulanic acid; TZP, piperacillin–tazobactam; TGC, tigecycline.

^*c*^ For CZA susceptibility testing, avibactam was used at a fixed concentration of 4 mg/L.

### Genomic evidence for within-clone evolution

**Fig 2:**
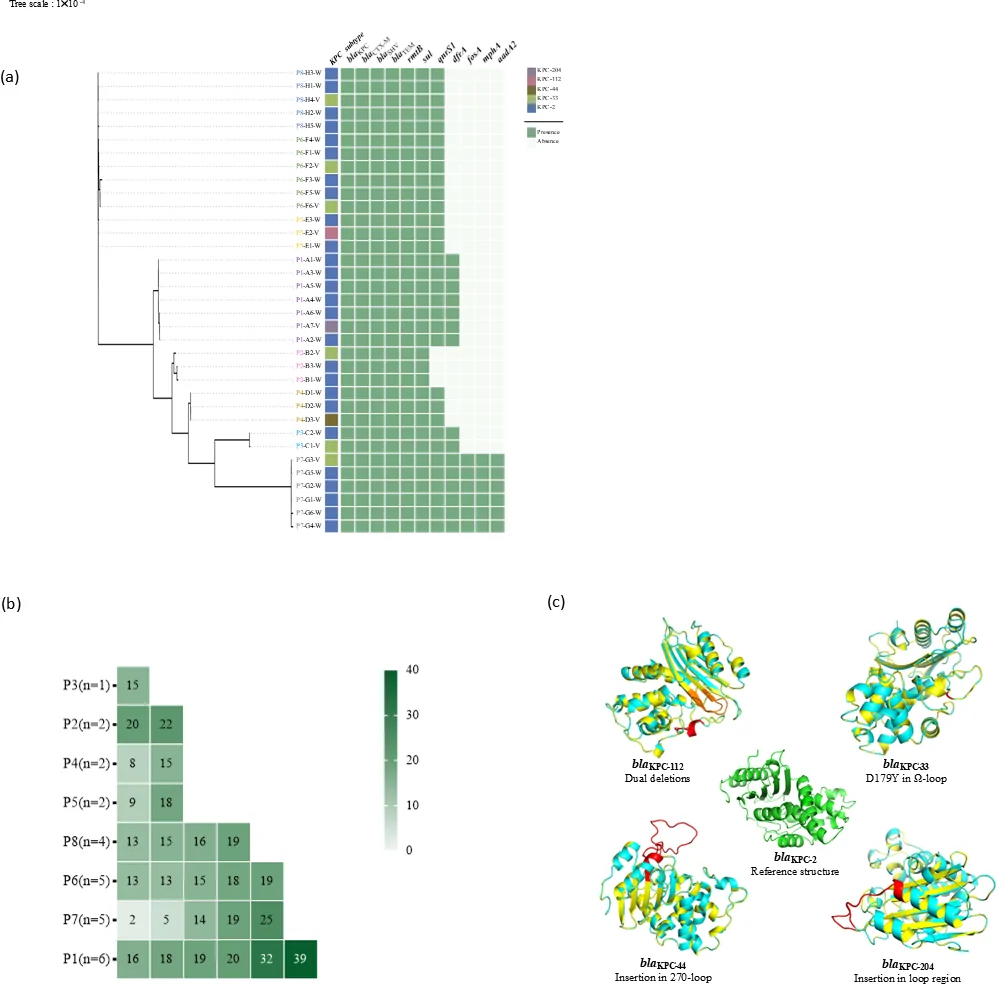
Phylogenetic, SNP, and structural analyses of KPC variants in ST11 *K. pneumoniae*. (**a**) Phylogenetic tree based on core-genome SNPs of the isolates. Strain labels are color-coded by patient origin; colored blocks indicate KPC subtypes; and the adjacent heatmap shows the presence or absence of major acquired antimicrobial resistance genes, including *bla*_KPC_, *bla*_CTX-M_, *bla*_SHV_, *bla*_TEM_, *rmtB*, *sul*, *qnrS1*, *dfrA*, *fosA*, *mphA*, and *aadA2*. Green blocks indicate gene presence, whereas light-colored blocks indicate gene absence. (**b**) Within-patient SNP distances between subsequent isolates and the first isolate recovered from the same patient. The numbers in each box indicate pairwise SNP differences, and the color scale represents the magnitude of divergence. *n* indicates the number of subsequent isolates recovered from each patient. (**c**) Structural comparison of KPC-2 and its variant proteins, highlighting the major functional loop regions harboring the mutations.

The phylogenetic tree constructed based on the core-genome single-nucleotide polymorphisms (SNPs) shows that all the isolates included in this study belong to the ST11 clonal type, but they are distributed across multiple branches. Isolates recovered from the same patient generally clustered closely on the phylogenetic tree, with within-patient SNP distances ranging from 2 to 39. The overall genetic distance heatmap based on core-genome comparisons likewise showed that isolates recovered from the same patient generally clustered within relatively compact low-divergence regions, whereas isolates from different patients exhibited greater overall divergence. (Fig. 2a; Fig. S1). Using the first isolate from each patient as a reference, we found that the subsequent isolates recovered from the same patient generally showed only small SNP differences relative to that index isolate (Fig. 2b), supporting the notion that these KPC variants arose through ongoing within-host evolution from pre-existing strain backgrounds.

For each KPC variant isolate and its corresponding clonally related KPC-2 isolate, the local genetic context surrounding *bla*_KPC_ was largely conserved, with no obvious large-scale rearrangements detected around the *bla*_KPC_ locus (Fig. S3). These isolates shared a similar Tn4401-associated *bla*_KPC_ genetic background, and the detected KPC variations were mainly confined to the coding sequence of *bla*_KPC_ itself. Amino acid sequence alignment showed that KPC-33 differed from KPC-2 by a D179Y substitution in the Ω-loop region, whereas KPC-44, KPC-112, and KPC-204 were characterized by an amino acid duplication in the 270-loop region, deletions involving two regions, and a loop insertion, respectively (Fig. S2 and Fig. 2c). These alterations were all located within or near known functional loop regions of the KPC-2 enzyme.

Based on genome annotation, we further compared porin status and the composition of major acquired antimicrobial resistance genes among longitudinal isolates from the same patients. Most isolates exhibited similar porin profiles, characterized by OmpK35-17% and OmpK36GD (Table S1). Heatmap analysis of major acquired antimicrobial resistance genes showed that longitudinal isolates from the same patient had consistent major acquired resistance gene profiles (Fig. 2a). Together, these findings indicate that KPC-2 isolates and KPC variant isolates from the same patient shared highly similar genetic backgrounds, with the principal differences concentrated in sequence variations within the *bla*_KPC_ coding region, consistent with within-host clonal evolution.

### Background-dependent fitness of KPC-33

**Fig 3:**
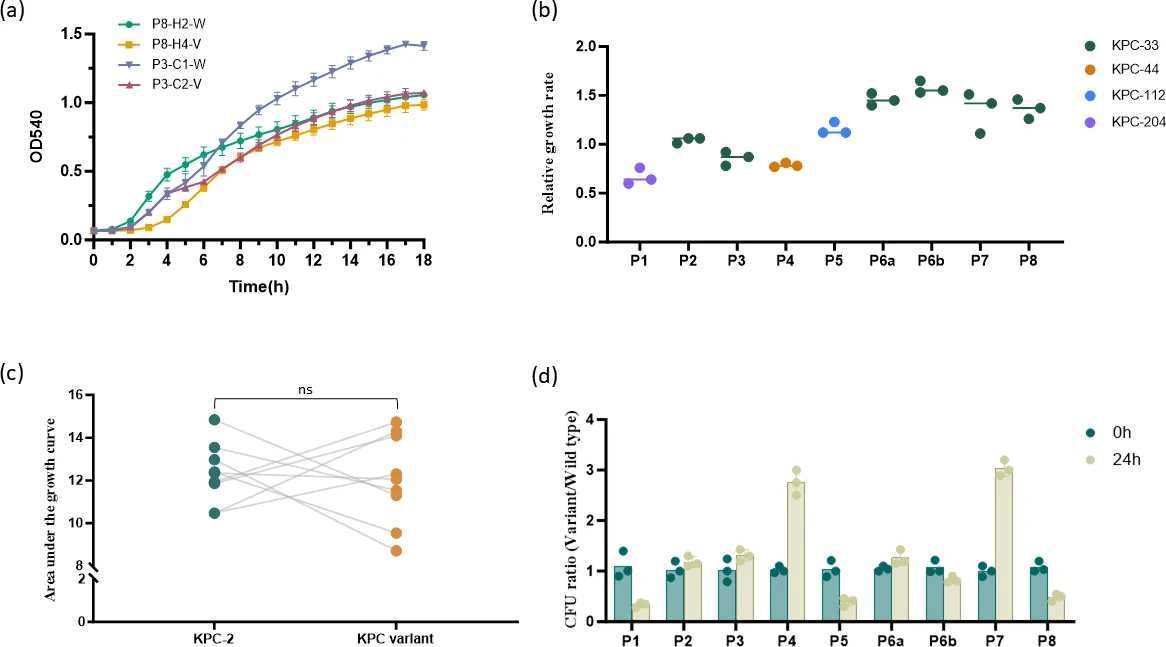
Growth and competitive phenotypes of KPC variants under antibiotic-free conditions. (**a**) Growth curves of a strain pair with competitive advantage (P3-C1-V/P3-C2-W) and a strain pair with competitive disadvantage (P8-H4-V/ P8-H2-W) under antibiotic-free conditions. (**b**) Relative growth rates of different KPC variants compared with their clonally related KPC-2 strains. Each point represents one strain, and the horizontal line indicates the mean value. (**c**) Paired comparison of the area under the growth curve between KPC-2 strains from each patient and their corresponding KPC variants. Each point represents the mean value of one patient-derived strain pair (*n* = 3), and lines indicate paired relationships. No statistically significant difference was observed between the two groups (Wilcoxon signed-rank test, *P* = 0.7344). (**d**) Results of the *in vitro* competition assay. The bar plot shows the CFU ratio of the variant strain to the wild-type strain at 0 and 24 h.

Growth curve analysis showed that, under antibiotic-free conditions, the overall growth patterns of the KPC variants were broadly similar to those of their corresponding parental KPC-2 strains (Fig. 3a; Fig. S4). Although some differences in growth rate and growth dynamics were observed among strains, particularly during the early growth phase, paired comparisons based on the area under the growth curve showed no statistically significant difference (Wilcoxon signed-rank test, *P* = 0.7344) (Fig. 3c), suggesting that acquisition of KPC variants did not lead to a consistent overall reduction in growth capacity.

*In vitro* competition assays showed that the fitness effects associated with resistance acquisition were not uniform across different KPC variants. Overall, the variants exhibited competition index (CI) changes that differed in both direction and magnitude during competition experiments. Further analysis revealed marked heterogeneity in the competitive phenotypes associated with KPC-33 across different strain backgrounds: in some strain pairs (P7-G3-V/P7-G2-W, P3-C1-V/P3-C2-W, P2-B2-V/P2-B1-W, and P6-F2-V/P6-F5-W), the variant-to-wild-type CFU ratio increased at 24 h relative to baseline, whereas in others (such as P6-F6-V/P6-F5-W and P8-H2-W/P8-H4-V), it decreased (Fig. 3d). Time-course competition assays further showed that this divergent trend was generally maintained over the 72 h observation period (Fig. S4). Notably, the two KPC-33 variants derived from patient P6 exhibited opposite competitive trajectories, further suggesting that the fitness effects of KPC-33 are strongly dependent on strain background.

Among the limited non-KPC-33 variants observed in this study, higher CZA resistance was not accompanied by a clearly favorable fitness profile compared with some KPC-33 backgrounds: KPC-44 showed relatively strong initial competitiveness, but its growth pattern was close to that of the wild type and its competition index declined over time. KPC-112 displayed a slight growth advantage, but its competitive ability also showed a downward trend. Although KPC-204 showed some improvement in the competition assay, it still carried a substantial overall fitness cost (Fig. 3d).

### Transcriptomic correlates of fitness heterogeneity

**Fig 4:**
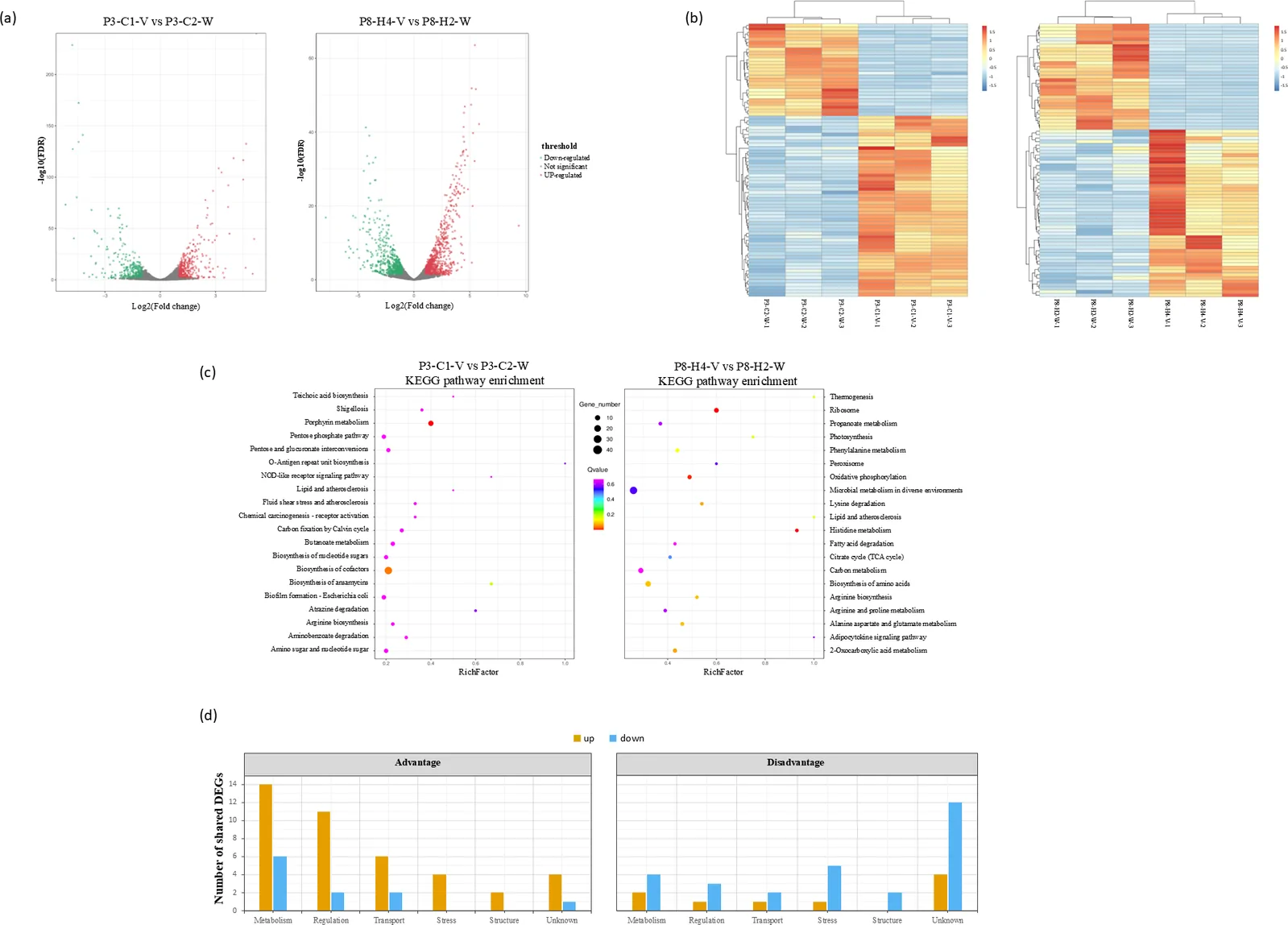
Comparative transcriptomic profiles of representative competitively advantageous and competitively disadvantaged KPC-33 strain pairs. (**a**) Volcano plots of differentially expressed genes (DEGs) in the competitively advantageous (P3-C1-V/P3-C2-W) and competitively disadvantaged (P8-H4-V/P8-H2-W). Each dot represents one gene. The *x*-axis indicates log_2_ fold change (log_2_FC), and the *y*-axis indicates −log_10_(FDR). (**b**) Clustered heatmap of differentially expressed genes in the two representative strain pairs described above. (**c**) Bubble plots showing KEGG pathway enrichment analysis of the two representative strain pairs. The *y*-axis indicates significantly enriched pathways. Bubble size represents the number of genes enriched in each pathway, and the color denotes the significance level (*Q* value). (**d**) Functional classification of shared DEGs in competitively disadvantaged and competitively advantageous strain pairs. The *y*-axis indicates the number of shared DEGs within each functional category. Shared DEGs in competitively advantageous and competitively disadvantaged strain pairs were predominantly downregulated overall, whereas those in competitively advantageous strain pairs were predominantly upregulated. Orange indicates upregulated genes, and blue indicates downregulated genes.

To explore the molecular basis of KPC-33-associated fitness heterogeneity, transcriptomic profiling was performed for all KPC-33 variants and their matched KPC-2 counterparts obtained from the same patients. The number of significantly differentially expressed genes varied markedly among the five paired isolate comparisons (Table S2). Among the competitively advantageous isolate pairs, 8, 718, 456, and 70 significantly differentially expressed genes were identified in P2-B2-V/P2-B1-W, P3-C1-V/P3-C2-W, and P6-F2-V/P6-F5-W, respectively. In contrast, 947 and 1,167 significantly differentially expressed genes were identified in the competitively disadvantaged pairs P6-F6-V/P6-F5-W and P8-H4-V/P8-H2-W, respectively. Overall, strain pairs with a competitive advantage generally exhibited more limited transcriptional changes, whereas competitively disadvantaged pairs tended to show more widespread differential expression (Fig. S6). To more clearly illustrate the transcriptomic features associated with these two phenotypic patterns, we further present a representative competitively advantageous pair, P3-C1-V/P3-C2-W, and a representative competitively disadvantaged pair, P8-H4-V/P8-H2-W (Fig. 4a through c).

In competitively advantageous KPC-33 strain pairs, the overall number of differentially expressed genes was relatively small, and the associated changes mainly involved functional categories related to transport, basic metabolism, and iron acquisition, although the specific patterns were not entirely consistent across strain pairs. The P3-C1-V/P3-C2-W strain pair was representative and showed a relatively small overall magnitude of transcriptional change. KEGG enrichment analysis showed that porphyrin metabolism (*Q* = 0.0015) and cofactor biosynthesis (*Q* = 0.0406) were significantly enriched. Genes related to iron uptake and storage, such as *fiu*, *efeO*, and *ftnA*, showed an upward trend. In contrast, genes involved in multiple transport systems, including PTS- and ABC transporter-related genes such as the *ptsJ*, *lsr*, and *liv* families, as well as the essential gene *lpxK*, were downregulated. In the P6-F2-V/P6-F5-W strain pair, KEGG enrichment analysis showed significant enrichment of the porphyrin metabolism pathway (*Q* = 4.31 × 10⁻⁵). Vitamin B12 biosynthesis-related genes, including *cbiE*, *cbiH*, *cobD*, *cobQ*, *cobU*, and *cobS*, showed an upward trend. In competitively disadvantaged KPC-33 strain pairs, the number of differentially expressed genes was generally higher. These genes were mainly related to substance transport, basic metabolism, and carbon source utilization. In the other competitively advantaged strain pairs, no significantly enriched pathways were identified (Fig. S6).

In low-fitness KPC-33 strains, the number of differentially expressed genes was relatively higher, and the range of transcriptional changes was broader. In the P8-H4-V/P8-H2-W strain pair, KEGG enrichment analysis showed significant enrichment of the ribosome, histidine metabolism, and oxidative phosphorylation pathways. Among these pathways, the ribosome pathway was the most significantly enriched (*Q* = 7.68 × 10⁻⁸), and multiple ribosomal protein-coding genes, such as *S4* and *S5*, showed an upward trend; some energy metabolism-related genes, such as succinate dehydrogenase and isocitrate dehydrogenase, showed a downward trend, and histidine metabolism-related genes also changed markedly. In the P6-F6-V/P6-F5-W strain pair, a broader pattern of transcriptional change was observed, including downregulation of the global transcriptional regulators *dksA* and *hns*, as well as the essential gene *acpP*. Genes related to the respiratory chain and hydrogen metabolism, such as the *nuo* and *hyf* families, were upregulated, together with several associated essential genes (e.g., *ceaC*, *rne*, *murF*, *murC*, and *murD*). Changes were also observed in genes related to transport systems and carbon metabolism (Fig. S6).

Further within-group intersection analysis supported these observations: competitively disadvantaged strain pairs shared a greater number of differentially expressed genes, predominantly downregulated, mainly involving functions related to transport, stress regulation, cell envelope/surface structure, and selected metabolic processes, whereas only a limited number of shared differentially expressed genes were detected among competitively advantageous strain pairs, and these were predominantly upregulated (Fig. 4d).

### Biochemical characterization of KPC-33

To further define the enzymatic consequences of the D179Y substitution, bla_KPC-2 and bla_KPC-33 from two longitudinal KPC-2/KPC-33 isolate pairs, P3-C2-W/P3-C1-V and P8-H2-W/P8-H4-V, were cloned, heterologously expressed, and purified. These independently derived enzymes were then used to assess the reproducibility of the enzymatic features associated with KPC-33 (Table 2). Hydrolysis curves for ceftazidime showed that, compared with the corresponding KPC-2 enzymes, KPC-33 exhibited consistently lower reaction rates (Fig. S7). In agreement with this hydrolysis pattern, KPC-33 enzymes from both isolate pairs showed lower *k*_cat_ and *K*_m_ values, whereas the calculated *k*_cat_/*K*_m_ values were overall comparable to or slightly lower than those of KPC-2. These results indicate that the D179Y substitution led to reproducible kinetic alterations in KPC-33, mainly characterized by reduced catalytic turnover and altered substrate interaction.

**TABLE 2:** Steady-state kinetic parameters of purified KPC-2 and KPC-33 enzymes against ceftazidime

Patient	Strain	Genotype	*k*_cat_ (s^−^¹)	*K*_m_ (μM)	*k*_cat_/*K*_m_ (μM^−^¹·s^−^¹)
P3	P3-C2-W	KPC-2	0.56 ± 0.05	520 ± 60	0.00108 ± 0.00016
	P3-C1-V	KPC-33	0.0142 ± 0.0008	14.3 ± 1.8	0.00099 ± 0.00014
P8	P8-H2-W	KPC-2	0.53 ± 0.04	495 ± 55	0.00107 ± 0.00014
	P8-H4-V	KPC-33	0.0112 ± 0.0007	15.6 ± 2.2	0.00072 ± 0.00011

## DISCUSSION

With the increasing clinical use of CZA for the treatment of infections caused by KPC-producing *K. pneumoniae*, the emergence of KPC variants has become an important issue in the evolution of CZA resistance (13, 14). Previous studies have reported that KPC-2-producing *K. pneumoniae* can give rise to various CZA resistance-associated KPC variants during CZA therapy or under related selective pressures (15, 16), with most studies focusing on how specific KPC variants alter enzymatic activity, reduce susceptibility to avibactam inhibition, or lead to increased CZA MICs (17, 18). However, resistance mutations alone are insufficient to explain the clinical emergence and persistence of KPC variants. Whether a resistance mutation can persist within a patient depends not only on the selective advantage it confers under antibiotic pressure (19), but also on its fitness consequences in a specific strain background. The central finding of this study is that KPC-33 does not impose a fixed fitness cost, but instead exhibits marked fitness heterogeneity across different ST11 genetic backgrounds, and this heterogeneity may be associated with differences in the global transcriptional state of the bacterial cell.

The recurrent detection of KPC-33 suggests that the D179Y substitution may represent a relatively reproducible evolutionary route under clinical antimicrobial selection pressure, especially in the setting of CZA exposure. Compared with other KPC variants involving larger insertions, deletions, or more complex structural alterations, KPC-33 is caused by a single amino acid substitution within the Ω-loop region (8, 10, 20, 21). This structural change appears sufficient to affect the CZA resistance phenotype, but may not necessarily impose severe global physiological impairment. Therefore, the clinical emergence of KPC-33 may reflect a relative balance between resistance benefit and fitness cost: although it may not confer the highest level of CZA resistance, it may provide sufficient resistance at a relatively low physiological cost in certain strain backgrounds.

More importantly, this study shows that the same KPC-33 variant can lead to different competitive outcomes across distinct longitudinal clonal backgrounds. This suggests that although changes in the *bla*_KPC_ sequence are key determinants of resistance development, their ultimate fitness effects are also shaped by the host strain background. Previous studies on the fitness costs of antibiotic resistance mutations have similarly shown that the fitness burden associated with resistance-related mutations is not fixed, but is often influenced by genetic background and environmental conditions (22, 23). Therefore, the clinical maintenance of KPC-33 should be interpreted in the context of both the strain background and the global physiological state of the bacterial cell. The detection frequency and persistence of different KPC variants in clinical settings may therefore depend not only on the level of resistance they confer, but also on the fitness costs they impose in specific strain backgrounds.

The enzyme kinetic results further support this interpretation. The D179Y substitution clearly alters the biochemical properties of the KPC enzyme and provides a direct enzymatic basis for CZA resistance (24). However, the enzymatic changes cannot fully explain the fitness differences among different KPC-33 isolates nor can they alone account for the relatively high carbapenem MICs observed in some isolates. Given that most isolates in this study exhibited porin features such as OmpK36GD insertion and OmpK35 truncation, the carbapenem MIC phenotype is more likely to result from the combined effects of KPC variation, porin status, and the ST11 genetic background, rather than being attributable solely to the D179Y substitution itself.

In other words, the “resistance” of KPC-33 can largely be understood at the enzyme level, whereas its “fitness” must be interpreted in the context of the whole-cell background. This represents an important distinction between the present study and studies focused solely on enzymology: we further demonstrate that clinical isolates carrying the same D179Y substitution can exhibit different fitness outcomes in different genetic backgrounds.

Transcriptomic analysis provides a possible explanation for this fitness difference. Overall, KPC-33 strains with better fitness did not appear to require extensive transcriptional reprogramming, whereas less fit strains were accompanied by broader transcriptional perturbations. This suggests that the fitness cost of KPC-33 may not arise entirely from the D179Y mutation itself, but may partly reflect the physiological adjustment required for the cell to accommodate this resistance mutation. In some strain backgrounds, limited metabolic or transport-related adjustments may be sufficient to maintain a relatively stable physiological state. In other backgrounds, however, acquisition of KPC-33 may trigger broader remodeling of energy metabolism, protein synthesis, transport systems, and envelope-related processes, reflecting insufficient compensation or increased physiological stress.

Based on these findings, we propose a possible explanatory model in which the maintenance of transcriptional homeostasis may influence the fitness outcome of KPC-33. When KPC-33 arises in a strain background capable of maintaining a relatively stable transcriptional state, its resistance benefit may be retained at a relatively low fitness cost. In contrast, when KPC-33 arises in a background that requires extensive transcriptional remodeling to maintain basic physiological functions, the strain may be more likely to exhibit a competitive disadvantage. Therefore, the fitness outcome of KPC-33 should be understood in the combined context of the resistance mutation, the strain background, and the global transcriptional state of the bacterial cell.

This finding also has potential clinical implications. For KPC variants emerging during CZA therapy, their risk should not be assessed solely on the basis of CZA MICs or KPC subtype. Even among isolates carrying the same KPC-33 variant, fitness outcomes may differ across strain backgrounds. Therefore, integrating resistance mutation detection with strain background, fitness status, and transcriptional state may help provide a more accurate understanding of the evolutionary risk of CZA-resistant strains within patients.

This study has several limitations. First, although it was based on longitudinal clinical isolates, the sample size remained limited, particularly for non-KPC-33 variants, which precluded a comprehensive comparison of fitness differences among different KPC variants. Second, our transcriptomic analysis suggests an association between the extent of transcriptional remodeling and the fitness phenotype; however, this evidence remains correlative and does not establish that any specific regulatory pathway directly determines the fitness outcome of KPC-33. Future studies using gene knockout, complementation, or overexpression approaches will be needed to further validate the causal roles of key regulators and downstream pathways. In addition, plasmid copy number, plasmid stability, plasmid genetic background, infection site, and host immune pressure may also influence the maintenance and competitive ability of KPC-33. Previous studies have suggested that specific plasmid backgrounds can affect the maintenance and dissemination potential of carbapenemase genes in *K. pneumoniae* (25). These issues should be further investigated in larger longitudinal clinical cohorts and functional studies.

To minimize genetic background differences other than the D179Y substitution, the KPC-2 parental isolates used for comparison in this study were selected from within the same patient as those with the closest core-genome SNP distances (2, 8, 9, 16, and 20 SNPs) to the corresponding KPC-33 variants. Nevertheless, it remains theoretically possible that accompanying mutations contribute to the observed phenotypic differences. However, longitudinal isolates from patient P6 provided a critical clue to distinguish between these two possibilities: the two KPC-33 variants sequentially isolated from this patient, despite sharing similar genetic backgrounds, displayed opposite competitive fitness—one advantageous and the other disadvantageous. If the direction of fitness were governed by accompanying mutations, longitudinally isolated strains sharing similar genetic backgrounds would be expected to converge toward similar fitness outcomes rather than exhibit opposite trajectories. Furthermore, a similar association pattern between fitness phenotypes and transcriptional remodeling was observed among KPC-33 isolates from multiple independent patients: strains with a competitive advantage typically exhibited limited transcriptional remodeling, whereas those with a competitive disadvantage displayed extensive transcriptional perturbations. This across-patient trend cannot be readily explained by independent random background mutations specific to each isolate. Collectively, D179Y emerges as the most likely major mutation responsible for the observed fitness heterogeneity, although its independent causal effect remains to be confirmed through allelic exchange experiments in an isogenic background.

In summary, this study demonstrates that KPC-33 can repeatedly emerge under CZA-associated clinical selective pressure, but its fitness consequences are not fixed. Rather than simply emphasizing D179Y-mediated CZA resistance, the more important finding of this study is that the same KPC-33 variant exhibits marked fitness heterogeneity across different ST11 strain backgrounds, and this heterogeneity may be associated with the ability of the strain to maintain a relatively stable transcriptional state. These findings suggest that the *in vivo* evolution of KPC variants should be understood not through a framework centered solely on individual resistance mutations but through an integrated framework involving resistance mutations, genetic background, and the global transcriptional state of the bacterial cell.

## MATERIALS AND METHODS

### Bacterial isolate collection and longitudinal patient monitoring

From June 2023 to June 2025, we conducted a longitudinal surveillance study of KPC-producing *K. pneumoniae* infections at a tertiary hospital in Jiangxi Province, China. The included KPC-producing *K. pneumoniae* infections were identified from clinically relevant specimens collected during routine care, including ascitic fluid, sputum, and secretion samples, corresponding mainly to intra-abdominal, respiratory, and secretion-related infections. Antimicrobial treatment information, including CZA exposure, was obtained from the corresponding medical records. During this period, all hospitalized patients with microbiologically confirmed KPC-producing *K. pneumoniae* infections were enrolled in the initial screening cohort. Through longitudinal sampling and isolate preservation in the initially screened patients, we ultimately identified eight patients who exhibited clear KPC genotype evolution during hospitalization and included them in the final analysis. All isolates were identified to the species level using a MALDI-TOF mass spectrometry system. Preliminary screening and genotyping of the *bla*_KPC_ gene were performed by polymerase chain reaction followed by Sanger sequencing.

### Antimicrobial susceptibility testing

MICs of all isolates were determined by broth microdilution. The antimicrobial agents tested included imipenem, meropenem, CZA (avibactam at a fixed concentration of 4 mg/L), ticarcillin–clavulanic acid, piperacillin–tazobactam, cefoperazone–sulbactam, ceftazidime, cefepime, aztreonam, amikacin, tobramycin, levofloxacin, ciprofloxacin, trimethoprim–sulfamethoxazole, doxycycline, minocycline, tigecycline, and colistin. Antimicrobial susceptibility results were interpreted according to the Clinical and Laboratory Standards Institute 2024 criteria (26), with colistin interpreted based on the European Committee on Antimicrobial Susceptibility Testing criteria. *Escherichia coli* ATCC 25922 was used as the quality control strain. For CZA selection assays, the ceftazidime concentration was set individually for each matched patient-derived pair as an intermediate concentration between the CZA MICs of the corresponding KPC-2-producing isolate and KPC variant-producing isolate, with avibactam fixed at 4 mg/L. At the same time, the corresponding KPC-2 isolate was plated on the same selective agar as a control, and no colony growth was observed.

### Whole-genome sequencing and bioinformatics analysis

All longitudinal isolates recovered from each patient, including both KPC-2-producing isolates and KPC variant isolates, were subjected to whole-genome sequencing. After quality assessment of genomic DNA, 100 ng of DNA from each sample was randomly fragmented using a Covaris S2 ultrasonicator (Covaris, USA) to generate DNA fragments with an average length of approximately 400 bp. Sequencing libraries were prepared from the fragmented DNA using the NEXTflex DNA Sequencing Kit (Bio Scientific, USA). After quality control, qualified libraries were subjected to high-throughput sequencing on the Illumina HiSeq X Ten platform. Raw reads were quality-filtered using Trimmomatic v0.39, and clean reads were assembled using Velvet v1.2.10. Gene prediction was performed using Prodigal, and functional annotation was performed against the NCBI nr, CDD, KEGG, VFDB, and CARD databases. SNP analysis was performed using Snippy v4.6.0, with *K. pneumoniae* HS11286 (GenBank: CP003200.1) as the reference genome. A maximum-likelihood phylogenetic tree was constructed from the core-genome SNP alignment and visualized and annotated using tvBOT (27). Porin alterations were assessed based on genome annotation results, with a primary focus on the OmpK status of the longitudinal isolates.

### *In vitro* growth curve and competition assays

To assess the fitness cost associated with KPC variants while minimizing the confounding effect of genetic background differences, the KPC-2 isolate with the smallest core-genome SNP distance to the corresponding KPC variant was selected as the control strain within each patient. These matched strain pairs were used for subsequent growth curve assays, *in vitro* competition experiments, transcriptomic analysis, and enzymatic assays. Each strain was tested in three independent biological replicates. Growth curves were generated by diluting overnight cultures to 10^6^ CFU/mL and inoculating them into 96-well plates containing fresh LB broth. Cultures were incubated at 37°C with continuous shaking for 20 h, and OD540 values were recorded every 5 min. The *in vitro* competition assay was performed by mixing overnight cultures of the wild-type and variant strains at equal volumes and inoculating the mixture into antibiotic-free LB broth, followed by incubation at 37°C with shaking. Samples were collected at 0, 24, 48, and 72 h and plated on CZA-containing selective plates and antibiotic-free LB agar plates to enumerate colonies of the variant and wild-type strains, respectively. The relative abundance of the variant and wild-type strains was expressed as the CFU ratio (variant/wild type), and the CI was further calculated as ln(variant CFU / wild-type CFU). All experiments were performed in three independent biological replicates, and the results are presented as the mean values.

### Transcriptomic analysis

To investigate transcriptional changes associated with the heterogeneous fitness effects of KPC-33, the same matched KPC-2/KPC variant isolate pairs used in the *in vitro* competition assays were subjected to RNA sequencing (28). Each strain was analyzed with three independent biological replicates. Bacterial cultures were grown in LB broth at 37°C with shaking and harvested during the exponential growth phase for total RNA extraction. Residual genomic DNA was removed by DNase I digestion, and RNA quality and integrity were assessed before library construction. For prokaryotic transcriptome sequencing, rRNA was depleted, and the remaining RNA was fragmented and used for strand-specific cDNA library preparation. Qualified libraries were sequenced on the Illumina NovaSeq 6000 platform. After quality filtering, clean reads were mapped to the corresponding reference genome using BWA. Gene expression levels were normalized as FPKM values. Differential expression analysis was performed using DESeq2 in Bioconductor, and genes with an FDR of ≤0.05 and a |log2Ratio| of ≥1 were defined as differentially expressed genes. KEGG pathway enrichment analysis was performed to characterize the functional distribution of differentially expressed genes.

### Protein expression and purification

To assess the enzymatic consequences of the D179Y substitution in KPC-33, two representative longitudinal isolate pairs, P3-C2-W/P3-C1-V and P8-H2-W/P8-H4-V, were selected for further analysis. The corresponding coding sequences of *bla*_KPC-2_ and *bla*_KPC-33_ were amplified and cloned into the pET-28a expression vector to generate recombinant proteins carrying an N-terminal His₆ tag. After sequence verification, the recombinant plasmids were introduced into *Escherichia coli* BL21(DE3) for heterologous expression. Positive clones were pre-cultured in LB medium supplemented with kanamycin and subsequently transferred into fresh medium for further growth. When the cultures reached the mid-logarithmic phase, recombinant protein expression was induced by the addition of IPTG. After induction, bacterial cells were harvested by centrifugation and disrupted by sonication to release soluble proteins. Cell debris was removed by centrifugation, and the resulting supernatant was used for protein purification. The crude enzyme preparation was first subjected to HiTrap Q HP anion-exchange chromatography for initial fractionation. Fractions containing the target protein were concentrated and further purified by size-exclusion chromatography using a HiLoad 16/600 Superdex 75 pg column. The purified proteins were used for subsequent steady-state kinetic assays. SDS-PAGE analysis confirmed the purity of the final protein preparations to be >95%, which were subsequently used for steady-state kinetic characterization.

### Enzyme activity and kinetic analysis

Ceftazidime was used as the representative substrate to compare the hydrolytic activities of purified KPC-2 and KPC-33 enzymes by ultraviolet-visible spectrophotometry. Reactions were performed in 1× PBS buffer at pH 7.4. A series of ceftazidime concentrations was tested, and changes in absorbance were continuously monitored at 260 nm during substrate hydrolysis. Absorbance changes were converted to product concentrations using the molar extinction coefficient of ceftazidime at 260 nm (Δε₂₆₀ = 8,660 M⁻¹·cm⁻¹). Initial reaction rates were determined at ceftazidime concentrations ranging from 10 to 1,000 μM, covering concentrations both below and above the *K*ₘ. Initial reaction rates were calculated from the linear phase of each reaction. The initial velocities obtained at different substrate concentrations were fitted to the Michaelis–Menten equation by non-linear regression using GraphPad Prism 10.1.2 to determine *K*_m_ and *k*cat values. Catalytic efficiency was expressed as *k*cat / *K*_m_. All experiments were performed in replicate, and data are presented as the mean ± standard deviation. The primers used for cloning are listed in Table S3.

## Data Availability

The genomic sequence data generated in this study have been deposited in the National Center for Biotechnology Information (NCBI) under BioProject accession no. PRJNA1456756. The associated BioSample and genome assembly records are publicly available under this BioProject. The RNA-seq raw sequencing data have been deposited in the NCBI Sequence Read Archive (SRA) under the same BioProject accession no. with SRA study accession no. SRP709493 and SRA run accession nos. SRR39144796–SRR39144828.

## SUPPLEMENTAL MATERIAL

Data S1 aac.00767-26-s0001.xlsx RNA-seq differential expression data for KPC-33 vs cognate KPC-2 strain pairs.

Supplemental legends aac.00767-26-s0002.docx Complete legends for the RNA-seq differential expression data (Data S1).

Supplemental material aac.00767-26-s0003.docx Fig. S1 to S7; Tables S1 to S3.

ASM does not own the copyrights to Supplemental Material that may be linked to, or accessed through, an article. The authors have granted ASM a non-exclusive, world-wide license to publish the Supplemental Material files. Please contact the corresponding author directly for reuse.
