# Supplementary material for: Repeated emergence and fitness heterogeneity of KPC-33 in ST11 Klebsiella pneumoniae under ceftazidime–avibactam pressure
Source: Antimicrob Agents Chemother. 2026 Sep 10;70(10):e00767-26. doi: 10.1128/aac.00767-26 (PMC13689381; doi:10.1128/aac.00767-26)
Supplement: Supplementary file 1 [file aac.00767-26-s0002.docx]

**Supplemental Data File.** Transcriptome annotation results for all KPC-33 vs. cognate KPC-2 strain pairs examined in this study.

This file contains RNA‑seq differential expression data for all KPC‑33 strains and their corresponding KPC‑2 parental strains. The strain pairs included are:

P2‑B2‑V vs. P2‑B1-W

P3-C2-V vs.P3-C1-W

P6‑F2‑V vs. P6‑F5‑W

P6‑F6‑V vs. P6‑F5‑W

P7‑G3‑V vs. P7‑G2‑W

P8‑H4‑V vs. P8‑H2‑W

This file contains the RNA‑seq differential expression data for each strain pair examined in this study. Detailed methods for RNA‑seq alignment and differential expression analysis are described in the Materials and Methods section of the main text. Genes with |log2Ratio| ≥ 1 and FDR < 0.05 were considered significantly differentially expressed.

**Column descriptions:**

**GeneID**: Gene identifier (locus tag or transcript ID)

**log2Ratio (KPC‑33 / KPC‑2)**: Log2 fold change in gene expression. Positive values indicate upregulation in KPC‑33; negative values indicate downregulation.

**Up‑Down‑Regulation**: Direction of regulation (up = upregulated in KPC‑33; down = downregulated).

**P‑value**: Statistical p‑value from the differential expression test.

**FDR**: False discovery rate adjusted p‑value (Benjamini–Hochberg correction). As noted above, "1" denotes that the gene passed the FDR < 0.05 cut‑off.
