# Supplementary material for: Repeated emergence and fitness heterogeneity of KPC-33 in ST11 Klebsiella pneumoniae under ceftazidime–avibactam pressure
Source: Antimicrob Agents Chemother. 2026 Sep 10;70(10):e00767-26. doi: 10.1128/aac.00767-26 (PMC13689381; doi:10.1128/aac.00767-26)
Supplement: Supplementary file 2 [file aac.00767-26-s0003.docx]

**Fig. S1. Heatmap of pairwise genetic distances among all longitudinal isolates based on core-genome comparison.**The heatmap shows the pairwise genetic distances among all longitudinal isolates included in this study based on core-genome comparison. Color intensity from light to dark indicates increasing genetic divergence, and isolates are ordered according to hierarchical clustering. Overall, isolates recovered from the same patient tended to cluster within relatively compact low-divergence regions, whereas isolates from different patients showed greater overall genetic divergence. This figure illustrates the global pattern of genetic relatedness among all isolates and provides supplementary support for the phylogenetic analysis presented in the main text.

**Fig. S2. Amino acid sequence alignment of different KPC variants and wild-type KPC.** Highlighted regions indicate amino acid differences.

**Fig. S3. Local genetic context surrounding *bla_KPC-2_* and its variant *bla*_KPC_ genes in representative longitudinal isolate pairs.** Homologous open reading frames are indicated by identical colors. The brackets indicate the *Tn4401*-associated region containing *bla*_KPC_. The *bla*_KPC_ flanking regions appeared largely conserved between paired KPC-2-producing isolates and their corresponding KPC variant-producing isolates.

**Fig. S4. Growth curves of all KPC variants and their corresponding clonally related KPC-2 strains under antibiotic-free conditions.** (a–c) KPC-33 strain pairs; (d) KPC-204 strain pair; (e) KPC-44 strain pair; (f) KPC-112 strain pair. Each curve represents the growth profile of the corresponding strain under antibiotic-free conditions. Data are presented as the mean of three independent biological replicates, and error bars indicate standard deviation.

**Fig. S5. Time-course in vitro competition assay results for all KPC variant strain pairs.** (a) Strain pairs showing an overall decrease in the competition index (CI).(b) Strain pairs showing an overall increase in the competition index (CI).The x-axis indicates incubation time, and the y-axis indicates the competition index, defined as CI = ln(variant CFU/wild-type CFU). The dashed line indicates CI = 0. Data are presented as the mean ± standard deviation of three independent biological replicates.

**Fig. S6. Transcriptomic profiles of the remaining competitively advantageous and competitively disadvantaged KPC-33 strain pairs.** (a–c) Volcano plots of differentially expressed genes (DEGs) and KEGG enrichment analysis of the remaining competitively advantageous strain pairs (P6-F2-V/P6-F5-W, P7-G3-V/P7-G2-W, and P2-B2-V/P2-B1-W). (d) Volcano plot of DEGs and KEGG enrichment analysis of the remaining competitively disadvantaged strain pair (P6-F6-V/P6-F5-W). In the volcano plots, the x-axis indicates log2FC and the y-axis indicates −log10(FDR). In the KEGG bubble plots, the y-axis indicates enriched pathways, the x-axis indicates the enrichment factor, bubble size represents the number of enriched genes, and color indicates the Q value.

**Supplementary Fig. S7. Ceftazidime hydrolysis progress curves of purified KPC-2 and KPC-33 enzymes.**Hydrolysis of ceftazidime (25 µM) by purified KPC-2 and KPC-33 enzymes (2 µM) was monitored by measuring the decrease in substrate concentration over time at 25°C. Compared with the corresponding KPC-2 enzymes (P3-C2-W and P8-H2-W), KPC-33 enzymes (P3-C1-V and P8-H4-V) exhibited slower ceftazidime hydrolysis. The no-enzyme control (CAZ only) remained stable over the experimental period.

**Supplementary Table S1**. OmpK35/OmpK36 porin status of longitudinal isolates

| Patient | Strains | KPC variant | OmpK35 status | OmpK36 status |
| --- | --- | --- | --- | --- |
| P1 | P1-A1-W | KPC-2 | OmpK35-17% | OmpK36GD |
|  | P1-A2-W | KPC-2 | OmpK35-17% | OmpK36GD |
|  | P1-A3-W | KPC-2 | OmpK35-17% | OmpK36GD |
|  | P1-A4-W | KPC-2 | OmpK35-17% | OmpK36GD |
|  | P1-A5-W | KPC-2 | OmpK35-17% | OmpK36GD |
|  | P1-A6-W | KPC-2 | OmpK35-17% | OmpK36GD |
|  | P1-A7-V | KPC-204 | OmpK35-17% | OmpK36GD |
| P2 | P2-B1-W | KPC-2 | OmpK35-17% | OmpK36GD |
|  | P2-B2-V | KPC-33 | OmpK35-17% | OmpK36GD |
|  | P2-B3-W | KPC-2 | OmpK35-17% | OmpK36GD |
| P3 | P3-C1-V | KPC-33 | OmpK35-17% | OmpK36GD |
|  | P3-C2-W | KPC-2 | OmpK35-17% | OmpK36GD |
| P4 | P4-D1-W | KPC-2 | OmpK35-17% | OmpK36GD |
|  | P4-D2-W | KPC-2 | OmpK35-17% | OmpK36GD |
|  | P4-D3-V | KPC-44 | OmpK35-17% | OmpK36GD |
| P5 | P5-E1-W | KPC-2 | OmpK35-17% | OmpK36GD |
|  | P5-E2-V | KPC-112 | OmpK35-17% | OmpK36GD |
|  | P5-E3-W | KPC-2 | OmpK35-17% | OmpK36GD |
| P6 | P6-F1-W | KPC-2 | OmpK35-17% | OmpK36GD |
|  | P6-F2-V | KPC-33 | OmpK35-17% | OmpK36GD |
|  | P6-F3-W | KPC-2 | OmpK35-17% | OmpK36GD |
|  | P6-F4-W | KPC-2 | OmpK35-17% | OmpK36GD |
|  | P6-F5-W | KPC-2 | OmpK35-17% | OmpK36GD |
|  | P6-F6-V | KPC-33 | OmpK35-17% | OmpK36GD |
| P7 | P7-G1-W | KPC-2 | OmpK35-17% | OmpK36GD |
|  | P7-G2-W | KPC-2 | OmpK35-17% | OmpK36GD |
|  | P7-G3-V | KPC-33 | OmpK35-17% | OmpK36GD |
|  | P7-G4-W | KPC-2 | OmpK35-17% | OmpK36GD |
|  | P7-G5-W | KPC-2 | OmpK35-17% | OmpK36GD |
|  | P7-G6-W | KPC-2 | OmpK35-17% | OmpK36GD |
| P8 | P8-H1-W | KPC-2 | OmpK35-17% | OmpK36GD |
|  | P8-H2-W | KPC-2 | OmpK35-17% | OmpK36GD |
|  | P8-H3-W | KPC-2 | OmpK35-17% | OmpK36GD |
|  | P8-H4-V | KPC-33 | OmpK35-17% | OmpK36GD |
|  | P8-H5-W | KPC-2 | OmpK35-17% | OmpK36GD |

**Supplementary Table S2.** Numbers of significantly differentially expressed genes in matched KPC-2/KPC variant isolate pairs

| Comparison | Fitness phenotype | Total DEGs | Upregulated | Downregulated |
| --- | --- | --- | --- | --- |
| P2-B1-W/P2-B2-V | Advantage | 8 | 3 | 5 |
| P3-C1-W/P3-C2-V | Advantage | 718 | 360 | 358 |
| P6-F5-W/P6-F2-V | Advantage | 456 | 222 | 234 |
| P6-F5-W/P6-F6-V | Disadvantage | 947 | 476 | 471 |
| P8-H2-W/ P8-H4-V | Disadvantage | 1167 | 648 | 519 |

**Supplementary Table S3.** Primers used for cloning of *bla*_KPC-2_ and *bla*_KPC-33_ into pET-28a.

| Primer | Sequence (5′–3′) |
| --- | --- |
| KPC-2-pET28a-F | TGGGTCGCGGATCCGAATTCATGTCACTGTATCGCCGTCTAGT |
| KPC-2-pET28a-R | TCGAGTGCGGCCGCAAGCTTTTACTGCCCGTTGACGCC |
| KPC-33-pET28a-F | TGGGTCGCGGATCCGAATTCATGTCACTGTATCGCCGTCTAGT |
| KPC-33-pET28a-R | TCGAGTGCGGCCGCAAGCTTTTACTGCCCGTTGACGCC |
